# Trends in 28-Day Mortality of Critical Care Patients With Coronavirus Disease 2019 in the United Kingdom: A National Cohort Study, March 2020 to January 2021*

**DOI:** 10.1097/CCM.0000000000005184

**Published:** 2021-07-13

**Authors:** John M. Dennis, Andrew P. McGovern, Nicholas J. Thomas, Harrison Wilde, Sebastian J. Vollmer, Bilal A. Mateen

**Affiliations:** 1 University of Exeter Medical School, Institute of Biomedical & Clinical Science, RILD Royal Devon & Exeter Hospital, Exeter, United Kingdom.; 2 Department of Statistics, University of Warwick, Coventry, United Kingdom.; 3 British Library, The Alan Turing Institute, London, United Kingdom.; 4 University College London, Institute of Health Informatics, London, United Kingdom.

**Keywords:** coronavirus infection, critical care, hospital mortality, public health surveillance, quality of healthcare

## Abstract

To determine whether the previously described trend of improving mortality in people with coronavirus disease 2019 in critical care during the first wave was maintained, plateaued, or reversed during the second wave in United Kingdom, when B117 became the dominant strain.

**DESIGN:**

National retrospective cohort study.

**SETTING:**

All English hospital trusts (i.e., groups of hospitals functioning as single operational units), reporting critical care admissions (high dependency unit and ICU) to the Coronavirus Disease 2019 Hospitalization in England Surveillance System.

**PATIENTS:**

A total of 49,862 (34,336 high dependency unit and 15,526 ICU) patients admitted between March 1, 2020, and January 31, 2021 (inclusive).

**INTERVENTIONS:**

Not applicable.

**MEASUREMENTS AND MAIN RESULTS:**

The primary outcome was inhospital 28-day mortality by calendar month of admission, from March 2020 to January 2021. Unadjusted mortality was estimated, and Cox proportional hazard models were used to estimate adjusted mortality, controlling for age, sex, ethnicity, major comorbidities, social deprivation, geographic location, and operational strain (using bed occupancy as a proxy). Mortality fell to trough levels in June 2020 (ICU: 22.5% [95% CI, 18.2–27.4], high dependency unit: 8.0% [95% CI, 6.4–9.6]) but then subsequently increased up to January 2021: (ICU: 30.6% [95% CI, 29.0–32.2] and high dependency unit, 16.2% [95% CI, 15.3–17.1]). Comparing patients admitted during June–September 2020 with those admitted during December 2020–January 2021, the adjusted mortality was 59% (CI range, 39–82) higher in high dependency unit and 88% (CI range, 62–118) higher in ICU for the later period. This increased mortality was seen in all subgroups including those under 65.

**CONCLUSIONS:**

There was a marked deterioration in outcomes for patients admitted to critical care at the peak of the second wave of coronavirus disease 2019 in United Kingdom (December 2020–January 2021), compared with the post-first-wave period (June 2020–September 2020). The deterioration was independent of recorded patient characteristics and occupancy levels. Further research is required to determine to what extent this deterioration reflects the impact of the B117 variant of concern.

## Abstract 2

Supplemental Digital Content is available in the text.

National data from the United Kingdom (1, 2), as well as internationally (3), suggest that the mortality risk for patients admitted to critical care settings with coronavirus disease 2019 (COVID-19) appears to have declined over time. This improvement is consistent with a growing evidence base on how to best manage patients with COVID-19, such as changes in ventilation strategy (4), identification of several effective pharmacological interventions (5, 6), and extending mechanical ventilation bed capacity to meet demand (7). However, near the end of 2020, new variants emerged, associated with increased rates of transmissibility (8, 9). These new variants in combination with the accompanying operational strain on health systems (10) raised concerns that mortality rates might again rise. In this study, we sought to assess whether the aforementioned trend of improving survival rates that we previously described in people with severe COVID-19 requiring critical care (high dependency unit [HDU] or ICU) management (1) was maintained during the second wave of COVID-19 in United Kingdom.

## MATERIALS AND METHODS

### Data Sources

Data pertaining to all adult COVID-19–specific critical care (HDU) and ICU) admissions across United Kingdom, between March 1, 2020, and January 31, 2021, were extracted from the COVID-19 Hospitalization in England Surveillance System (CHESS)—a surveillance dataset containing information on all individuals with diagnostic test confirmed or clinically presumed COVID-19 managed in HDU or ICU (11). Follow-up data were available until March 5, 2021. Daily trust-level bed occupancy data (from April 1, 2020, to January 31, 2021, March 2020 data were not available) were extracted from the daily situation reports submitted by each trust to the national regulator (12).

### Individual-Level Critical Care Data

The following characteristics were extracted for each individual from CHESS: age, sex, ethnicity, first segment of postcode (used to identify the relevant indices of multiple deprivation for the corresponding areas in United Kingdom), admitting hospital trust (each trust may comprise more than one hospital), and recorded comorbidities (obesity, diabetes, asthma, other chronic respiratory disease, chronic heart disease, hypertension, immunosuppression due to disease or treatment, chronic neurologic disease, chronic renal disease, and chronic liver disease). We coded ethnicity as: White, Asian, Black, mixed, and other, categorized hospital centers by region: London, East of United Kingdom, Midlands, North East and Yorkshire, North West, South East, and South West, and recorded comorbidities as binary No/Yes variables. Missing data were assumed to represent the absence of comorbidity and the appropriateness of this imputation procedure, and alternatives are explored elsewhere (13).

### Occupancy Data

Trust-level occupancy of HDU beds and beds compatible with mechanical ventilation (as a proxy for ICU strain [7]) were linked to each individual’s record based on their admitting trust. Occupancy was defined as a percentage, relative to capacity during the baseline prepandemic period (January–March 2020). We linked information from daily situation reports on prepandemic (January–March 2020) number of beds compatible with mechanical ventilation, the number of HDU beds, the proportion of beds compatible with mechanical ventilation occupied on each day of the study period, and each trust’s geographical region. Linkage was carried out based on the trust that an individual was admitted to and the date of admission in CHESS; patients in CHESS were matched via their admission date to the relevant occupancy information from the corresponding date in the daily situation reports. The full preparation and linking of these data to CHESS are reported elsewhere (10).

### Eligibility and Study Cohorts

Patients 18–99 yr were eligible, but pregnant women (*n* = 430) were excluded. Subsequently, two cohorts were defined, the first comprising all people admitted to HDU but not ICU, and the second including all people admitted to ICU.

### Statistical Analysis

The primary outcome was inhospital all-cause mortality in the 28 days after hospital admission for HDU admitted patients and 28 days after intensive care admission for ICU patients. Patients discharged alive or transferred prior to 28 days were assumed to be alive at 28 days. We estimated absolute (unadjusted) mortality for each calendar month (March 2020 to January 2021 inclusive) as the proportions of deaths/total number of admissions, overall and for subgroups defined by age (less than/greater than or equal to 65) and recorded comorbidity (none/one or more). Adjusted mortality by calendar month of admission (categorical variable) was estimated using Cox proportional hazards models, adjusting for age (three-knot nonlinear restricted cubic spline), sex, ethnicity, recorded comorbidities, deprivation index, and geographical region, with proportional hazards assumptions tested. For the ICU cohort, we additionally adjusted for the number of days from hospital to ICU admission (to capture possible changes in admission policy over time, e.g., if there were delays in admitting patients to ICU in months when concern over hospital capacity was greatest). As sensitivity analysis, we repeated the overall model adjusting for occupancy and with hospital trust included as a random effect. To further explore mortality during the second wave in United Kingdom, we grouped admissions in the months of December 2020–January 2021 (peak second wave), early second wave (October–November 2020), post-first wave (June 2020–September 2020), and first wave (March–April 2020) and compared adjusted (Cox models) mortality across periods, overall and for age and comorbidity-defined subgroups. Analyses were conducted with R (Version 3.6.2; R Foundation for Statistical Computing, Vienna, Austria [14]), including the following packages: survival, rms and coxme.

## RESULTS

**Figure 1.**
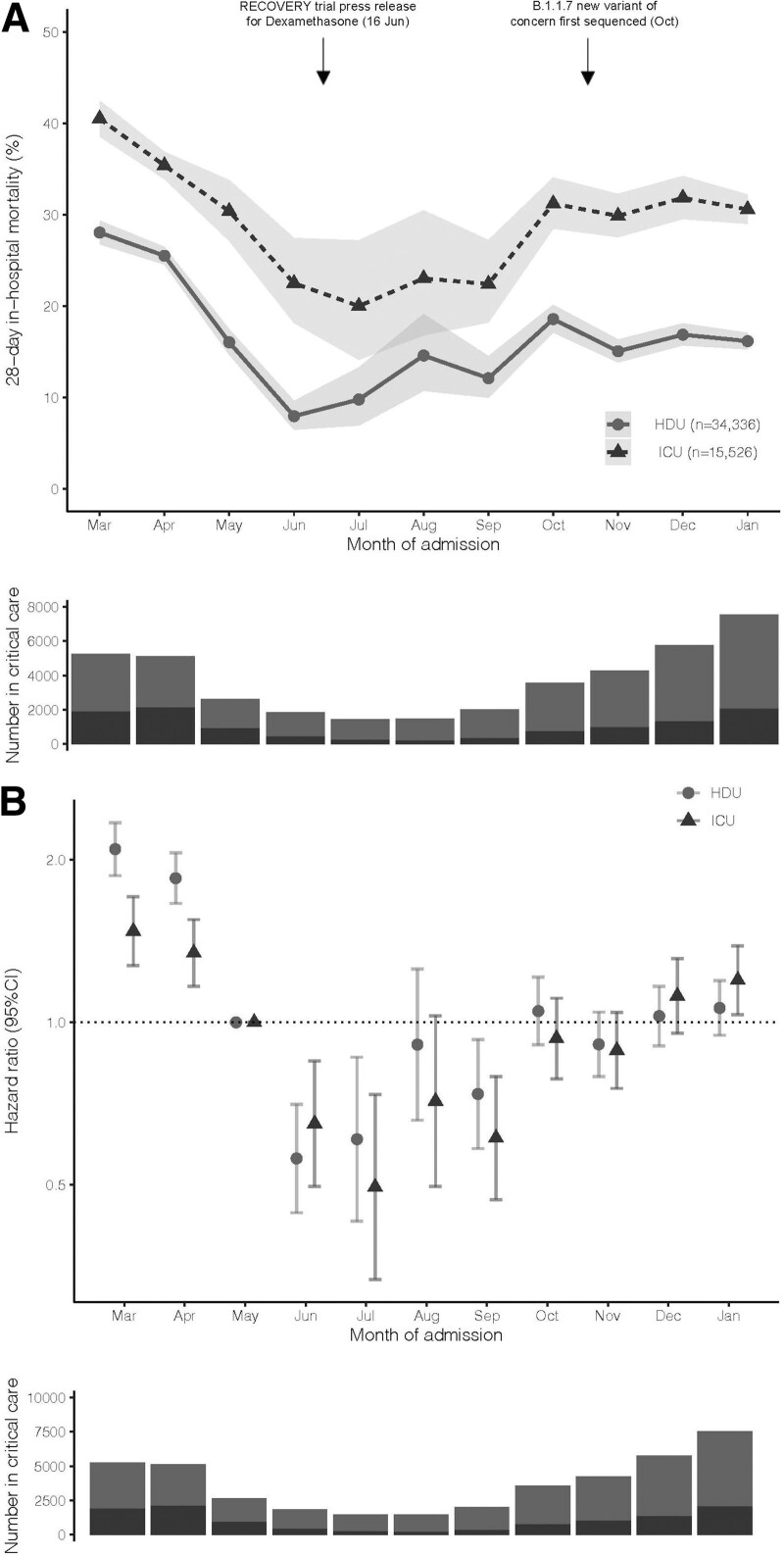
Mortality risk and number of admissions by month from March 2020 to January 2021. **A**, Number of patients in critical care and 28-d inhospital mortality, by calendar month. Underlying numerical data are provided in **sTable 2** (http://links.lww.com/CCM/G595). **B**, Adjusted mortality by calendar month. Estimates are hazard ratios representing the difference in mortality relative to May 2020, chosen as the month after the peak of the first wave, but prior to the Randomized Evaluation of COVID-19 Therapy (RECOVERY) press release demonstrating the efficacy of dexamethasone (5). Note: adjustments are for age (three knot restricted cubic spline), sex, ethnicity, recorded comorbidities, deprivation index, and geographical region. Bars represent 95% CIs. HDU = high dependency unit.

A total of 49,862 (34,336 HDU and 15,526 ICU) patients were included across 110 hospital trusts (**sFlowchart**, http://links.lww.com/CCM/G595); 6,765 (19.7%) HDU and 5,119 (32.9%) ICU patients died within 28 days of admission. Mean follow-up was 24 days (sd, 8 d) for HDU patients and 20 days (sd, 10 d) for ICU patients. Recorded characteristics are reported in **sTable 1** (http://links.lww.com/CCM/G595); patients admitted to ICU were older (mean 70 [sd, 17] vs 59 [sd, 13]), were more commonly male (68.8% vs 53.5%), and had greater recorded major comorbidity burden (43.6% vs 39.6% with at least one major comorbidity). The number of patients in critical care peaked in January 2021 (**Fig. 1*A***).

Twenty-eight-day inhospital mortality decreased from March 2020 to June 2020: ICU March 40.5% (95% CI, 38.6–42.5), June 22.5% (95% CI, 18.2–27.4); HDU March 28.0% (95% CI, 26.8–29.3), June 8.0% (95% CI, 6.5–9.6), before increasing up to January 2021: ICU 30.6% (95% CI, 29.0–32.2), HDU 16.2% (95% CI, 15.3–17.1) (Fig. 1*A*). Differences persisted following adjustment for recorded characteristics (**Fig. 1*B***).

For patients admitted in December 2020–January 2021 (peak second wave) compared with June–September 2020 (post-first wave), adjusted mortality was 88% (CI, 62–118) higher in ICU and 59% (CI, 39–82) higher in HDU (sTable 2, http://links.lww.com/CCM/G595). The mortality increase was greater in the peak second wave than that in the early second wave (October–November 2020), but never reached the mortality level of the first wave (March–April 2020) (**sTable 3**, http://links.lww.com/CCM/G595). Adjusted mortality differences by time period were similar across subgroups defined by age and comorbidity, including patients under 65 (**Table 1**; **sFig. 1** and sTable 3, http://links.lww.com/CCM/G595).

Results were concordant with the primary analysis when adjusting for occupancy level on the day of admission as an additional covariate (available for 28,414 HDU and 12,099 ICU patients), and with admitting trust modeled as an additional random effect (Table 1; and **sFig. 2**, http://links.lww.com/CCM/G595].

**TABLE 1.** Overall and Subgroup Analysis to Compare Outcomes of Patients Admitted During June–September 2020 (Post-First-Wave Period) Versus December 2020–January 2021 (Peak of the Second Wave in the United Kingdom)

Patient Characteristics	June–September Absolute Mortality (%)	December–January 2021 Absolute Mortality (%)	Difference (% [CI])	Unadjusted HR (95% CI)	Adjusted HR (95%CI)^b^	Occupancy Adjusted HR (95% CI)^c^
High dependency unit						
Overall	268/2,628 (10.2%)	1,689/10,285 (16.4%)	6.2 (4.8–7.6)	1.66 (1.46–1.89) *p* < 0.001	1.59 (1.39–1.82), *p* < 0.001	1.35 (1.17–1.56), *p* < 0.001
Patients < 65 yr	21/923 (2.3%)	129/3,515 (3.7%)	1.4 (0.2–2,6)	1.61 (1.02–2.56), *p* = 0.04	1.41 (0.86–2.31), *p* = 0.17	1.31 (0.77–2.23), *p* = 0.32
Patients ≥ 65 yr	247/1,706 (14.5%)	1,560/6,770 (23.0%)	8.6 (6.6–10.5)	1.67 (1.46–1.90), *p* < 0.001	1.52 (1.33–1.74), *p* < 0.001	1.35 (1.16–1.58), *p* < 0.001
Patients without recorded major comorbidity^a^	136/1,641 (8.3%)	989/6,780 (14.6%)	6.3(4.7–7.9)	1.81 (1.52–2.17), *p* < 0.001	1.40 (1.17–1.69), *p* < 0.001	1.15 (0.93–1.40), *p* = 0.19
Patients with at least one recorded major comorbidity^a^	132/988 (13.4%)	700/3,505 (20.0%)	6.6(4.0–9.2)	1.54 (1.28–1.86), *p* < 0.001	1.45 (1.20–1.77), *p* < 0.001	1.45 (1.17–1.80), *p* < 0.001
ICU						
Overall	213/961 (22.2%)	1,480/4,776 (31.0%)	8.8(5.8–11.8)	1.58 (1.37–1.83), *p* < 0.001	1.88 (1.62–2.18), *p* < 0.001	1.71 (1.45–2.01), *p* < 0.001
Patients < 65 yr	89/613 (14.5%)	684/3,018 (22.7%)	8.1(4.9–14.5)	1.74 (1.39–2.17), *p* < 0.001	2.04 (1.62–2.57), *p* < 0.001	1.85 (1.44–2.36), *p* < 0.001
Patients ≥ 65 yr	124/348 (35.6%)	796/1,758 (45.3%)	9.6(4.5–15.4)	1.50 (1.24–1.81), *p* < 0.001	1.70 (1.40–2.07), *p* < 0.001	1.56 (1.26–1.93), *p* < 0.001
Patients without recorded major comorbidity^a^	62/400 (15.5%)	784/2,918 (26.9%)	11.4(7.3–15.4)	1.98 (1.53–2.56), *p* < 0.001	2.06 (1.58–2.69), *p* < 0.001	1.93 (1.46–2.55), *p* < 0.001
Patients with at least one recorded major comorbidity^a^	151/561 (26.9%)	696/1,858 (37.5%)	10.5(6.1–14.9)	1.63 (1.37–1.95), *p* < 0.001	1.84 (1.53–2.21), *p* < 0.001	1.64 (1.34–2.00), *p* < 0.001

HR = hazard ratio.

^a^No diabetes, chronic respiratory disease, heart disease, renal disease, liver disease, neurologic disease, or immunosuppession.

^b^Adjusted for age (three knot restricted cubic spline), sex, ethnicity, recorded comorbidities, deprivation index, and geographical region.

^c^Adjusted for occupancy, age (three knot restricted cubic spline), sex, ethnicity, recorded comorbidities, deprivation index, and geographical region. Occupancy available only for patient subset (high dependency unit overall, *n* = 28,414; aged less than 65, *n* = 9,119; aged greater than or equal to 65, *n* = 19,295; no major recorded comorbidity, *n* = 17,207; major comorbidity, *n* = 11,207; ICU: overall, *n* = 12,099; aged less than 65, *n* = 7,665; aged greater than or equal to 65, *n* = 4,434; no major recorded comorbidity, *n* = 6,876; comorbidity, *n* = 5,223).

## DISCUSSION

Our study of nearly 50,000 critical care patients illustrates a marked deterioration in outcomes for patients admitted at the peak of the second wave of COVID-19 pandemic in the United Kingdom (December 2020–January 2021) compared with the post-first-wave period (June–September 2020). In absolute terms when comparing the peak of the second-wave to the post-first-wave period (June–September 2020), mortality was 8.8% higher in ICU and 6.2% higher in HDU. Notably, despite this increase, peak second-wave mortality never reached that seen during the first wave.

The overall trend we observed in CHESS is concordant with those reported by the United Kingdom’s national critical care audit Intensive Care National Audit & Research Centre (ICNARC) that collected critical care data via a separate mechanism (15). Both analyses show 28-day mortality has increased since the post-first-wave period while never reaching the first-wave peak of March–April 2020, the period prior to the Randomized, Embedded, Multi-factorial, Adaptive Platform Trial for Community-Acquired Pneumonia (REMAP-CAP) and Randomized Evaluation of COVID-19 Therapy (RECOVERY) trial results demonstrating efficacious therapies (6, 7). A novel finding of our study is that the deterioration in patient outcomes in recent months has been observed in younger as well as older adults, and in people with and without major comorbidity. These findings highlight the importance of continuing population-level disease suppression strategies until younger, healthier people have been vaccinated as well as the at-risk and elderly.

Further research is needed to understand the causes of the mortality time trends observed. This is likely multifactorial, with potential influences including: changes in the severity of critical care patients, health system operational strain, and the emergence of the new B117 variants of concern (VOCs) in the United Kingdom, which was first sequenced in October 2020 (16) and became the dominant strain in individuals who tested positive for COVID-19 by November 2020. Of note, when comparing post-B117 (December–January 2021) to pre-B117 (June–September 2020) time periods, we show a 59% (95% CI, 39–82) higher mortality in HDU and 88% (95% CI, 62–108) higher mortality in ICU, which is concordant with the reported 64% (95% CI, 32–104) higher mortality associated with the B117 variant in the United Kingdom compared with other strains of severe acute respiratory syndrome coronavirus 2 (8). Although we lacked data on severity of illness at admission to critical care, congruence of our results with ICNARC analysis, which includes these physiologic data, suggests residual confounding by disease severity is unlikely to explain our findings (15). In terms of precritical care treatment, dexamethasone was consistently and widely used to treat people with COVID-19 presenting to hospital in the United Kingdom over both the summer period and second wave. This consistent treatment pattern means that patients with severe treatment-refractory infection requiring critical care are likely to be comparable across the two time periods, although we lacked information on treatment and total COVID-19 hospitalizations to interrogate this further. Notably, ours is the first analysis to adjust for operational strain and demonstrate it did not explain mortality time trends, although this may not be a comprehensive proxy, as “unsafe” occupancy levels reflect a small minority of all operational issues that hospitals reported during the pandemic (B. A. Mateen et al, unpublished observations, 2021), and data for several thousand people admitted during March 2020 are missing, as these data was not available for that time period (see sFlowchart, http://links.lww.com/CCM/G595). The other major operational risk factor that is yet to have its impact characterized during the pandemic is that of staff absence and burnout, an important question for future research.

## CONCLUSIONS

The second wave of COVID-19 in United Kingdom saw critical care mortality rates deteriorate over December 2020–January 2021 to levels markedly higher than those observed in the post-first-wave period of June–September 2020. Further research is needed to determine to what extent this deterioration reflects the impact of the B117 VOC.

## Supplementary Material


