# Supplementary material for: Trends in 28-Day Mortality of Critical Care Patients With Coronavirus Disease 2019 in the United Kingdom: A National Cohort Study, March 2020 to January 2021*
Source: Crit Care Med. 2021 Jul 13;49(11):1895–900. doi: 10.1097/CCM.0000000000005184 (PMC8507592; doi:10.1097/CCM.0000000000005184)
Supplement: Supplementary file 1 [file ccm-49-1895-s001.docx]

**Supplementary material for:** Trends in 28-day mortality of critical care patients with COVID-19 in England: A national cohort study, March 2020 to January 2021

**Authors:** John M. Dennis, Andrew P. McGovern, Nick J Thomas, Harrison Wilde, Sebastian J. Vollmer, Bilal A. Mateen

**sFlowchart: Application of inclusion and exclusion criteria to generate final samples**


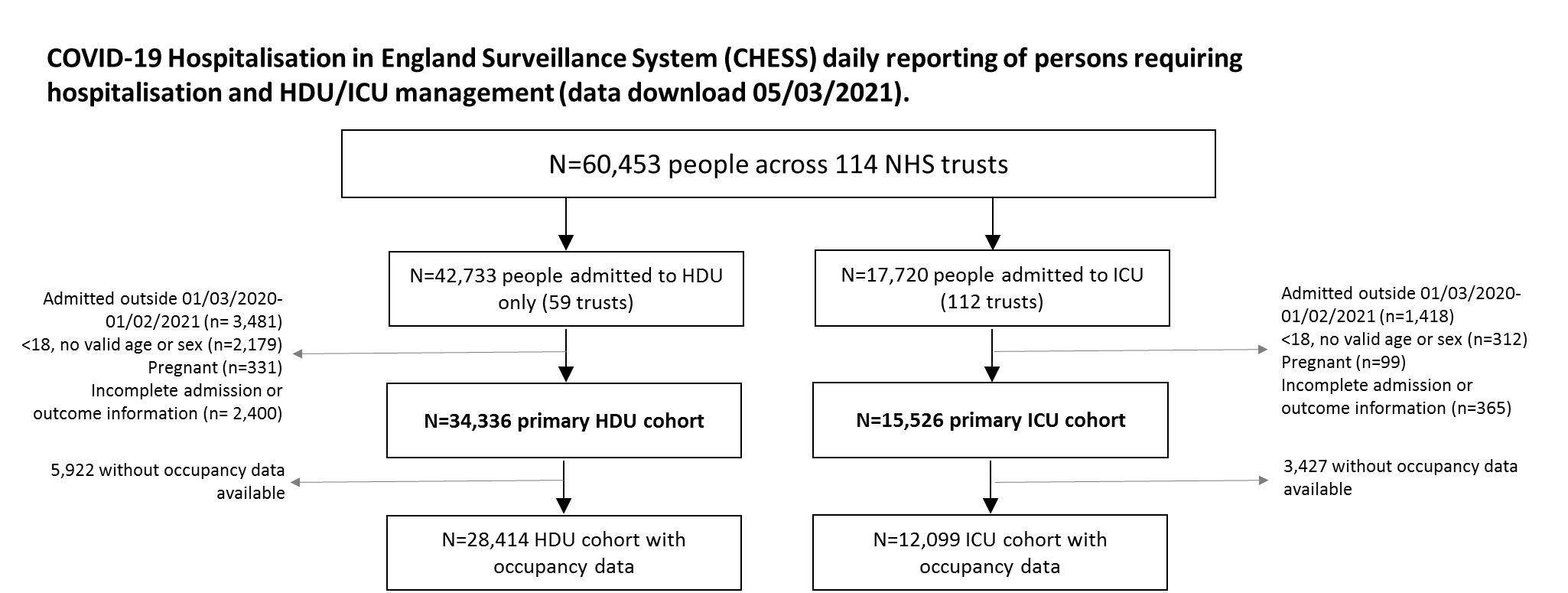


**sTable 1: Recorded characteristics of HDU and ICU cohorts.** Data are N (%) unless stated.

|  | **HDU (n=** **34,336)** | **ICU (n=15,526)** |
| --- | --- | --- |
| **Age (mean [SD])** |  |  |
|  | 70 (17) | 59 (13) |
| **Sex** |  |  |
| Female | 15,978 (46.5) | 4,833 (31.1) |
| Male | 18,358 (53.5) | 10,693 (68.8) |
| **Ethnicity*** | | |
| White | 24,871 (83.8) | 9058 (66.6) |
| Asian | 2,269 (7.6) | 2,309 (17.0) |
| Black | 1,376 (4.6) | 992 (7.3) |
| Mixed | 183 (0.6) | 264 (1.9) |
| Other | 994 (3.3) | 980 (7.2) |
| **Obesity*** | | |
| Non-obese | 13,415 (82.8) | 5,266 (46.3) |
| Obese | 2,781 (17.2) | 6,104 (53.7) |
| **IMD decile (mean [SD])** |  |  |
|  | 5.2 (2.0) | 5.2 (2.1) |
| **Comorbidity** | | |
| At least one major comorbidity† | 13,611 (39.6) | 6,767 (43.6) |
| Diabetes | 5,030 (14.6) | 3,814 (24.6) |
| Chronic renal disease | 3,278 (9.5) | 1,110 (7.1) |
| Chronic respiratory disease | 3,450 (10.0) | 1,467 (9.4) |
| Chronic heart disease | 5,799 (16.9) | 1,785 (11.5) |
| Chronic liver disease | 477 (1.4) | 348 (2.2) |
| Chronic neurological disease | 3,863 (11.3) | 592 (3.8) |
| Immunosuppressive disease | 907 (2.6) | 432 (2.8) |
| Asthma | 2367 (6.9) | 1,857 (12.0) |
| Hypertension | 8,935 (26.0) | 5,040 (32.5) |
| **Region*** |  |  |
| East of England | 6,525 (19.0) | 3,796 (24.4) |
| London | 5,579 (16.2) | 1,058 (6.8) |
| Midlands | 7,451 (21.7) | 2,051 (13.2) |
| North East & Yorkshire | 1,893 (5.5) | 2,270 (14.6) |
| North West | 7,132 (20.8) | 2,153 (13.9) |
| South East | 3,437 (10.0) | 2,147 (13.8) |
| South West | 1,473 (4.3) | 1,171 (7.5) |

*Ethnicity not recorded for 4,643 HDU patients and 1,923 ICU patients. Obesity not recorded for 18,140 HDU patients and 4,156 ICU patients. Region/IMD not available for 846 HDU patients and 880 ICU patients. †At least one of: diabetes, chronic respiratory disease, heart disease, renal disease, liver disease, neurological disease or immunosuppession

**sTable 2: Number of patients admitted and 28-day in-hospital mortality, by calendar month from March 2020-January 2021.**

**A) HDU**

| **Week of admission** | **Number of patients admitted** | **Number of patients dying within 28 days of admission** | **Mortality %**  **(95% CI)** |
| --- | --- | --- | --- |
| March 2020 | 4724 | 1325 | 28.0 (26.8, 29.3) |
| April | 8147 | 2078 | 25.5 (24.6, 26.5) |
| May | 2775 | 445 | 16.0 (14.7, 17.4) |
| June | 1182 | 94 | 8.0 (6.5, 9.6) |
| July | 348 | 34 | 9.8 (6.9, 13.3) |
| August | 281 | 41 | 14.6 (10.7, 19.1) |
| September | 818 | 99 | 12.1 (10.0, 14.5) |
| October | 2568 | 477 | 18.6 (17.1, 20.1) |
| November | 3208 | 483 | 15.1 (13.8, 16.3) |
| December | 3806 | 642 | 16.9 (15.7, 18.1) |
| January 2021 | 6479 | 1047 | 16.2 (15.3, 17.1) |

**B) ICU**

| **Month of admission** | **Number of patients admitted** | **Number of patients dying within 28 days of admission** | **Mortality %**  **(95% CI)** |
| --- | --- | --- | --- |
| March 2020 | 2441 | 989 | 40.5 (38.6, 42.5) |
| April | 4058 | 1436 | 35.4 (33.9, 36.9) |
| May | 747 | 227 | 30.4 (27.1, 33.8) |
| June | 320 | 72 | 22.5 (18.2, 27.4) |
| July | 150 | 30 | 20.0 (14.1, 27.2) |
| August | 152 | 35 | 23.0 (16.8, 30.5) |
| September | 339 | 76 | 22.4 (18.2, 27.2) |
| October | 1070 | 334 | 31.2 (28.5, 34.1) |
| November | 1473 | 440 | 29.9 (27.5, 32.3) |
| December | 1523 | 485 | 31.8 (29.5, 34.2) |
| January 2021 | 3253 | 995 | 30.6 (29.0, 32.2) |

**sFigure 1: 28-day in-hospital mortality (% of admitted patients), by calendar month, for age and comorbidity defined subgroups**

**
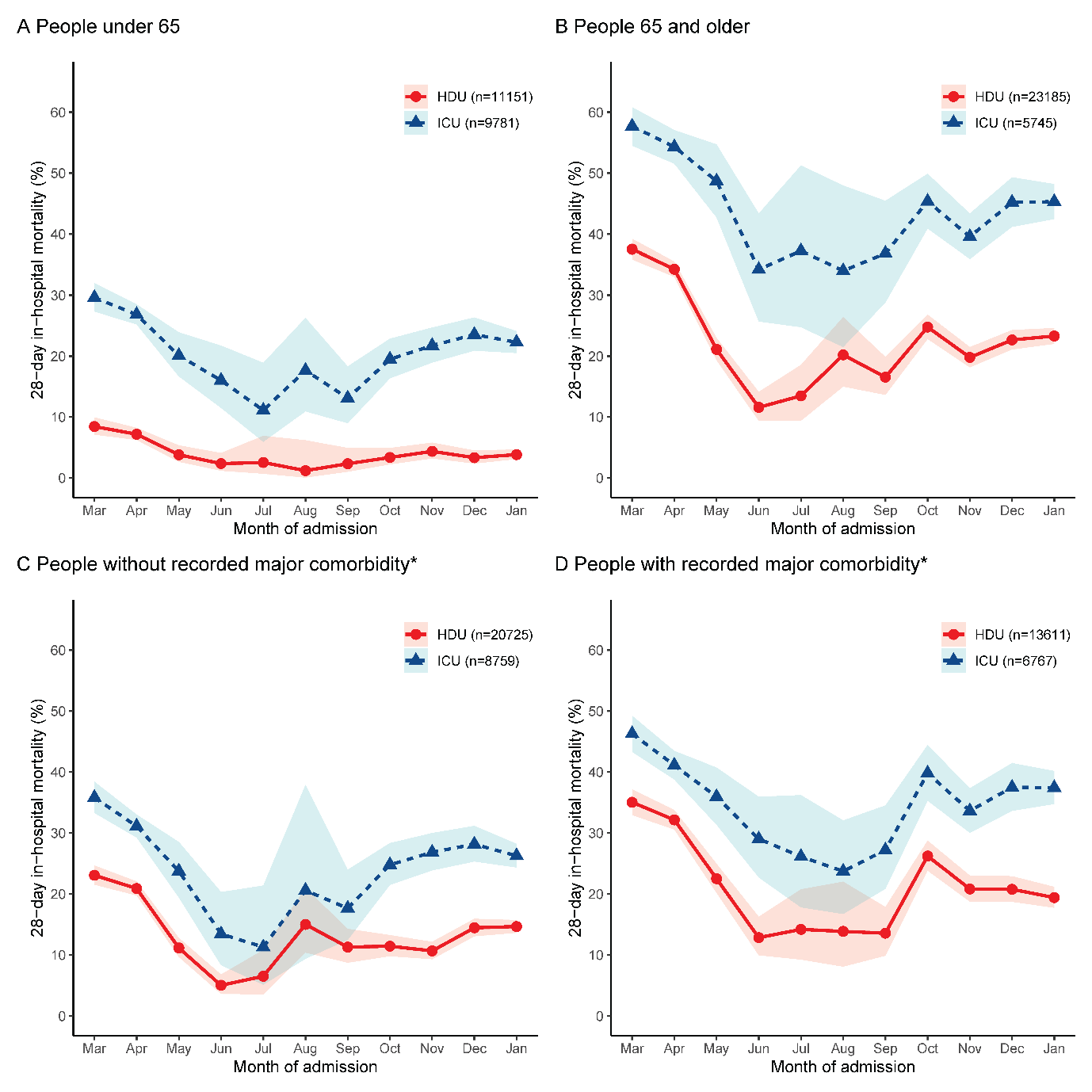
**

**sTable 3: Overall and subgroup analysis to compare adjusted mortality of patients admitted during June-September 2020 (post first wave period) versus first wave period (March-May 2020), early second wave period (October-November 2020) and peak second wave period (December 2020-January 2021).** Data are adjusted hazard ratios [aHR] (95% confidence intervals), adjusted for age (3 knot restricted cubic spline), sex, ethnicity, recorded comorbidities, deprivation index, and geographical region.

**a) HDU**

|  | **Mar-May 2020**  **aHR (95% CI)** | **Jun-Sep 2020**  **(reference group)** | **Oct-Nov 2020**  **aHR (95% CI)** | **Dec 2020-Jan 2021**  **aHR (95% CI)** |
| --- | --- | --- | --- | --- |
| **Overall** | 2.58 (2.26, 2.94) | 1.00 (ref) | 1.46 (1.26, 1.68) | 1.59 (1.39, 1.82) |
| **Subgroups** |  |  |  |  |
| **Age <65** | 3.47 (2.17, 5.56) | 1.00 (ref) | 1.21 (0.71, 2.05) | 1.41 (0.86, 2.31) |
| **Age ≥65** | 2.51 (2.19, 2.87) | 1.00 (ref) | 1.48 (1.27, 1.71) | 1.60 (1.39. 1.84) |
| **Without major comorbidity*** | 2.38 (1.98, 2.86) | 1.00 (ref) | 1.23 (1.00, 1.51) | 1.54 (1.28, 1.86) |
| **With major comorbidity** | 2.42 (2.10, 2.78) | 1.00 (ref) | 1.33 (1.14, 1.56) | 1.50 (1.30, 1.73) |

**b) ICU**

|  | **Mar-May 2020**  **aHR (95% CI)** | **Jun-Sep 2020**  **(reference group)** | **Oct-Nov 2020**  **aHR (95% CI)** | **Dec 2020-Jan 2021**  **aHR (95% CI)** |
| --- | --- | --- | --- | --- |
| **Overall** | 2.15 (1.86, 2.49) | 1.00 (ref) | 1.46 (1.25, 1.70) | 1.88 (1.62, 2.17) |
| **Subgroups** |  |  |  |  |
| **Age <65** | 2.22 (1.77, 2.78) | 1.00 (ref) | 1.64 (1.29, 2.10) | 2.04 (1.62, 2.57) |
| **Age ≥65** | 2.07 (1.71, 2.51) | 1.00 (ref) | 1.31 (1.07, 1.61) | 1.70 (1.40, 2.07) |
| **Without major comorbidity*** | 2.06 (1.58, 2.69) | 1.00 (ref) | 1.31 (1.07, 1.61) | 2.07 (1.71, 2.51) |
| **With major comorbidity** | 1.90 (1.59, 2.27) | 1.00 (ref) | 1.36 (1.12, 1.65) | 1.84 (1.53, 2.21) |

**sFigure 2: Adjusted mortality by calendar month for sensitivity analysis A) adjusting for occupancy, B) adjusting for hospital trust as a random effect.*** Estimates are hazard ratios representing the difference in mortality relative to May 2020, chosen as the month after the peak of the first wave, but prior to the RECOVERY press release demonstrating the efficacy of Dexamethasone. Point estimates are adjusted hazard ratios, additionally adjusted for age (3 knot restricted cubic spline), sex, ethnicity, recorded comorbidities, deprivation index, and geographical region. Bars represent 95% confidence intervals.

**
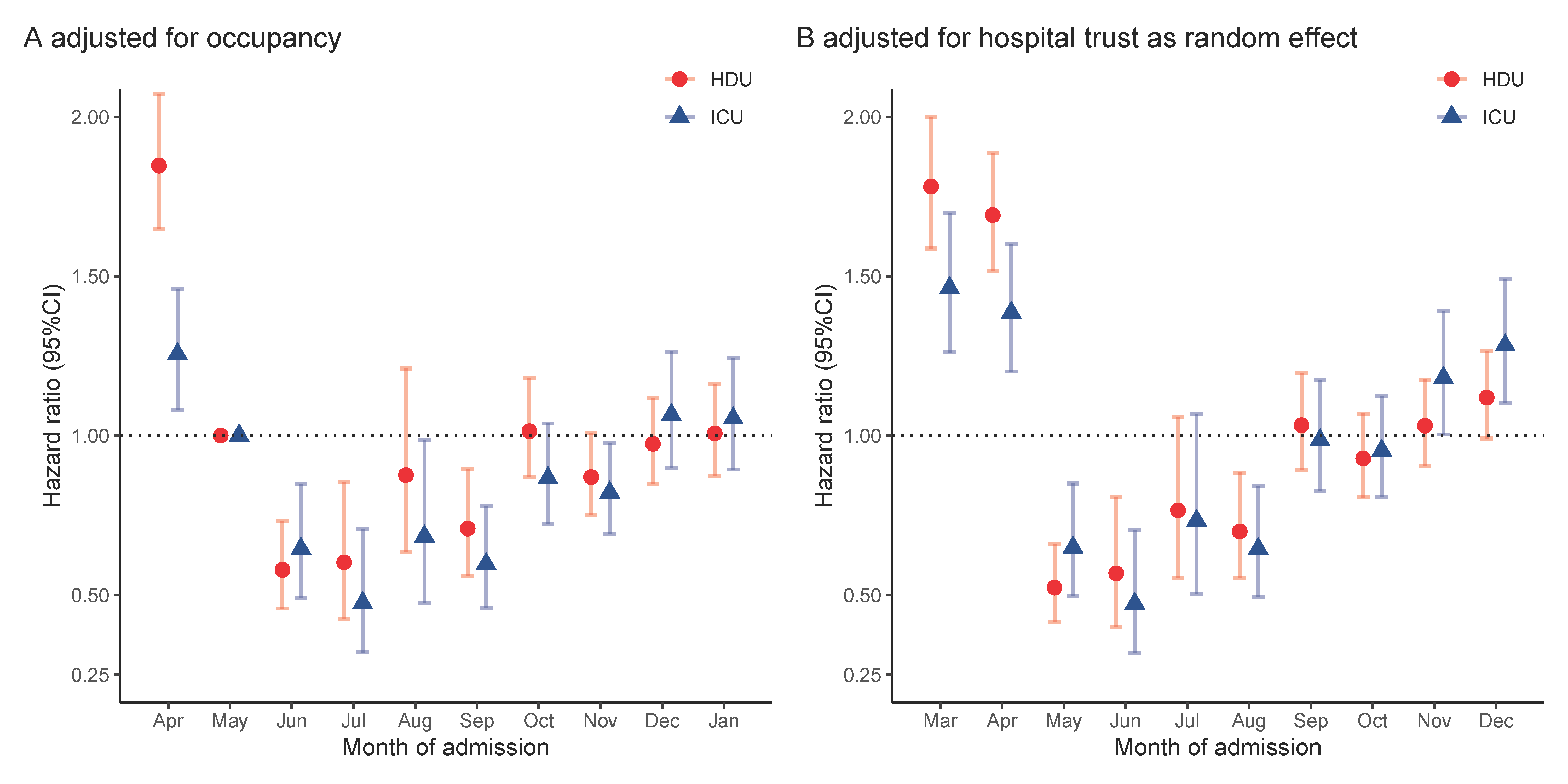
**
